# Longitudinal PET studies of mGluR5 in FXS using an FMR1 knockout mouse model

**DOI:** 10.1515/tnsci-2022-0217

**Published:** 2022-04-28

**Authors:** Sepideh Afshar, Sevda Lule, Gengyang Yuan, Xiying Qu, Chuzhi Pan, Michael Whalen, Anna-Liisa Brownell, Maria Mody

**Affiliations:** Gordon Center for Medical Imaging, Department of Radiology, Massachusetts General Hospital, Harvard Medical School, Charlestown, 02129 MA, United States of America; Department of Pediatrics, Massachusetts General Hospital, Harvard Medical School, Charlestown, 02129 MA, United States of America; Athinoula A. Martinos Center for Biomedical Imaging, Department of Radiology, Massachusetts General Hospital, Harvard Medical School, Charlestown, 02129 MA, USA

**Keywords:** FXS, positron emission tomography, [^18^F]FPEB, metabotropic glutamate receptor 5, mouse model

## Abstract

Fragile X syndrome (FXS) is a monogenic disorder characterized by intellectual disability and behavioral challenges. It is caused by aberrant methylation of the fragile X mental retardation 1 (FMR1) gene. Given the failure of clinical trials in FXS and growing evidence of a role of metabotropic glutamate subtype 5 receptors (mGluR5) in the pathophysiology of the disorder, we investigated mGluR5 function in FMR1 Knockout (FMR1-KO) mice and age- and sex-matched control mice using longitudinal positron emission tomography (PET) imaging to better understand the disorder. The studies were repeated at four time points to examine age- and disease-induced changes in mGluR5 availability using 3-fluoro-[^18^F]5-(2-pyridinylethynyl)benzonitrile ([^18^F]FPEB). We found that the binding potential (BP) of [^18^F]FPEB was significantly lower in the KO mice in mGluR5-implicated brain areas including striatum, cortex, hippocampus, thalamus, and olfactory bulb. The BP also changed with age, regardless of disorder status, increasing in early adulthood in male but not in female mice before decreasing later in both sexes. The difference in mGluR5 availability between the FMR1-KO and control mice and the change in BP in the KO mice as a function of age and sex illustrate the nature of the disorder and its progression, providing mechanistic insights for treatment design.

## Abbreviations

FXS: fragile X syndrome
FMR1: fragile X mental retardation 1
FMRP: fragile X mental retardation protein
PET: positron emission tomography
mGluR5: metabotropic glutamate subtype 5 receptor
FMR1-KO: FMR1 Knockout
GABA: gamma-aminobutyric acid
MLEM: maximum-likelihood expectation-maximization
CT: computed tomography
BP_ND_
: binding potential calculated based on reference tissue
G-protein: guanine nucleotide-binding protein
qq plot: nonparametric techniques to compare two batches of data
R: programming language
ID/cc: percent of the injected activity per cm^3^

## 1 Introduction

Fragile X syndrome (FXS) is a monogenic developmental disorder caused by mutation of the fragile X mental retardation 1 (FMR1) gene. The silenced FMR1 gene leads to suppression of the fragile X mental retardation protein (FMRP) [1]. As such, individuals with FXS fail to produce normal levels of the FMRP, which leads to intellectual disability and other neurological symptoms.

According to the mGluR theory of FXS, absence of FMRP regulatory control leads to increased protein synthesis and exaggerated metabotropic glutamate subtype 5 receptor (mGluR5) signaling, affecting relative glutamate and gamma-aminobutyric acid (GABA) levels creating excitatory–inhibitory neurotransmitter imbalance [2,3,4]. This results in deficits in neurogenesis and synaptic maintenance [5], leading to the formation of excessive long and thin dendritic spines resembling immature cortical networks [6,7,8,9], and alterations in cortical neural circuitry [10]. As a member of the G-protein-coupled receptor family, mGluR5 is expressed postsynaptically and is thought to account for multiple cognitive and syndromic features of FXS [3]. In fact, findings from FMR1 Knockout (FMR1-KO) mice and genetic deletion studies support the hypothesis that aberrant regulation of mGluR5 can lead to developmental synaptic disorders such as FXS or autism spectrum disorder and hence can serve as a target for interventions.

To understand mGluR5 function and how related glutamate transmission in the brain is affected in individuals with FXS, it is essential to investigate receptor availability *in vivo*. To this end, positron emission tomography (PET) imaging can be used with mGluR5-targeted radiolabeled ligands to assess binding potential (BP), an index of receptor availability. However, there are only a few studies of mGlu5 receptors in humans with FXS, and findings related to mGluR5 expression in animals and autopsies of humans with FXS have been inconsistent [4]. Promising findings from preclinical studies using drugs to block mGluR5 have failed to generalize to human subjects in clinical trials, raising questions about our understanding of the impaired mGluR5 mechanism in FXS.

The FMR1-KO mouse was the first animal model used to study FXS and has been the model of interest in many studies over 20 years [11]. In this model, the FXS phenotype is recapitulated by deletion of the FMR1 gene. The FMR1-KO mouse has many of the clinical features of FXS, both physiological and behavioral [12], that have been widely used to assess and track response to different treatments for the disorder [13,14,15,16,17]. Whereas behavioral tests provide valuable metrics of symptom severity for tracking progression of the disorder, we focus here on neuroimaging methods such as PET imaging, which allow us to probe the underlying neurobiological mechanism for potential use in clinical trials.

Of relevance here is the mGluR5 theory of FXS proposed by Bear and colleagues [2]. To this end, we used 3-fluoro-[^18^F]5-(2-pyridinylethynyl) benzonitrile ([^18^F]FPEB) [18], a compound with high specificity and binding affinity for mGluR5 to assess the glutamatergic neurosystem in an FMR1-KO mouse model. The PET imaging with [^18^F]FPEB was conducted at four different ages in FMR1-KO and control mice to compare mGlu5 receptor availability and changes with age and sex. To the best of our knowledge, we are among the first to investigate the effects of age and gender on mGluR5 function in the context of FXS, as indexed by receptor availability in FMR1-KO mice using a longitudinal study design. The overarching objective of this study is to gain insights from *in vivo* imaging of mGluR5 in the FMR1-KO mouse model toward improving the design and outcomes of clinical trials with FXS.

## 2 Materials and methods

### 2.1 Experimental animals and study design

A total of 56 mice, 28 FMR1-KO mice (B6.129P2-Fmr1tm1Cgr/J) and 28 age- and gender-matched control mice (C57BL/6NJ) comprising 14 male and 14 female mice in each group, were purchased from Jackson Laboratories, Maine, at 4 weeks of age and housed in groups of four per cage, of the same sex and genetic background, under standardized conditions. For identification of the mice, the tails were color-coded. The mice underwent acclimatization training; however, 20% of them were lost to aggression and in-fighting during a year-long research period. The remaining 44 mice (24 FMR1-KO mice: 12 male and 12 female; 20 control: 9 male and 11 female) underwent PET scanning at four ages: A1, A2, A3, and A4 (see Table 1) designed to examine the effects of age and sex on mGluR5 function in FMR1-KO mice. Due to equipment failure, not all mice were scanned at A2. See Table 1 for complete details.

**Table 1:** Different ages (A1–A4, in days) at which the FMR-1 and control mice underwent PET scanning

	A1 (days)	A2* (days)	A3 (days)	A4 (days)
FMR1-KO	43–57	91–100	156–219	325–398
	(M = 12, F = 12)	(M = 8, F = 8)	(M = 12, F = 12)	(M = 12, F = 12)
Control	41–51	84–93	156–216	325–395
	(M = 9, F = 11)	(M = 8, F = 7)	(M = 9, F = 11)	(M = 9, F = 11)

M = male; F = female. *Smaller number of mice is due to equipment failure issues.


**Ethical approval:** This study was approved and conducted under the guidelines of the Subcommittee on Research Animals of the Massachusetts General Hospital and Harvard Medical School in accordance with the NIH Guidelines for the Care and Use of Laboratory Animals.

### 2.2 PET imaging

In preparation for PET imaging, a mouse was first anesthetized with isoflurane/oxygen (1.5–2% isoflurane with oxygen flow of 2 L/min), and the tail vein was catheterized for bolus injection of the radioligand before being positioned into the micro-PET scanner (Triumph II, Trifoil Imaging, Inc.). The animal head was secured in a plexiglass custom-made head holder designed to ensure reproducible head positioning. The level of anesthesia and vital signs were monitored throughout the imaging procedures using the physiological monitoring system included with the imaging device.

Starting from the administration of [^18^F]FPEB (150–200 µCi in 20–30 µL i.v.; the specific activity at the time of injection 1,950–2,100 mCi/µmol), dynamic volumetric imaging data were acquired for 45 min, followed by a computed tomography (CT) scan to obtain data for attenuation correction and anatomical registration of the PET data.

After correction for uniformity, scatter, and attenuation, the PET data were processed using maximum-likelihood expectation-maximization algorithm provided by the manufacturer. Dynamic volumetric images with a spatial resolution of about 1 mm (9 × 20″, 7 × 60″ and 7 × 300″) were obtained after 30 iterations. The CT data were processed using the modified Feldkamp algorithm with a 512 × 512 × 512 matrix volume and pixel size of 170 μm. Co-registration of CT and PET images and analysis of PET data were implemented with PMOD 3.2 software (PMOD Technology, Zurich, Switzerland).

Six regions of interest (ROIs), *viz*., striatum, cortex (i.e., average cortex area), hippocampus, thalamus, hypothalamus, and olfactory bulb known to be implicated in FXS [19,20,21,22], were rendered on coronal and axial slices by aligning borders of different brain areas, using the Allen mouse brain atlas, with the fused CT-PET images. Time-activity curves (TACs) were extracted from the selected brain areas in the units of percent activity of the injected dose in the unit volume of cm^3^ or mL (% ID/cc), and BP_ND_ was calculated for each area from the TACs within the 25–45 min time window using cerebellum as a reference tissue.

### 2.3 Statistical analysis

We examined the effects of disorder status, age, and sex on [^18^F]FPEB BP in the FMR1-KO mice and age- and sex-matched control mice in the selected ROIs. The data were inspected for normality using qq-plot as well as for linearity and homogeneity of the residuals using residual plots. Linear mixed model analyses were conducted, with age as a within-subjects variable and disorder status and sex as between-subjects variables, in each brain area. Main and significant interaction effects were followed up by *post-hoc t* tests to further interrogate the group differences (Tables 2–6; Figures 2–4). Findings were corrected for multiple comparison (*p* < 0.05, Bonferroni-corrected for multiple brain regions and *post-hoc* tests). All the statistical analysis were done in R.

**Table 2:** Linear mixed model effects of disorder status, age, and sex in different brain areas

Variables	Striatum	Cortex	Hippocampus	Thalamus	Hypothalamus	Olfactory bulb
Disorder	9.081	16.416	34.477	16.246	2.307	11.463
F(1,149)	*p* = 0.004	*p* = 0.0002	*p* = 6.3 × 10^−7^	*p* = 0.0002	*p* = 0.131	*p* = 0.002
Sex	8.582	2.218	2.875	1.201	1.443	1.591
F(1,149)	*p* = 0.004	*p* = 0.139	*p* = 0.092	*p* = 0.275	*p* = 0.232	*p* = 0.209
Age	72.854	58.769	93.104	51.268	25.618	25.733
F(3,147)	*p* < 2.2 × 10^−16^	*p* < 2.2 × 10^−16^	*p* < 2.2 × 10^−16^	*p* < 2.2 × 10^−16^	*p* = 3.7 × 10^−13^	*p* = 1.6 × 10^−12^
Disorder × Sex	0.062	0.121	0.567	1.105	0.152	1.189
F(1,149)	*p* = 0.804	*p* = 0.728	*p* = 0.453	*p* = 0.295	*p* = 0.697	*p* = 0.277
Disorder × Age	0.230	1.475	1.371	1.307	2.224	0.825
F(3,147)	*p* = 0.875	*p* = 0.226	*p* = 0.256	*p* = 0.276	*p* = 0.088	*p* = 0.483
Sex × Age	5.231	0.944	4.895	1.627	3.443	0.347
F(3,147)	*p* = 0.002	*p* = 0.422	*p* = 0.003	*p* = 0.187	*p* = 0.019	*p* = 0.792
Disorder × Sex × Age	0.658	0.429	0.159	0.704	1.165	0.260
F(3,147)	*p* = 0.579	*p* = 0.732	*p* = 0.924	*p* = 0.551	*p* = 0.326	*p* = 0.854

**Table 3:** [^18^F]FPEB BPs in Control and FMR1-KO mice, regardless of age and sex in selected brain areas

	BP_ND_	
Brain area	Control	FMR1-KO
Striatum	4.896 ± 1.237	4.474 ± 1.021
Cortex	4.843 ± 1.530	4.154 ± 1.168
Hippocampus	5.585 ± 1.516	4.733 ± 1.262
Thalamus	3.247 ± 0.653	2.924 ± 0.594
Hypothalamus	2.905 + 0.499	2.753 ± 0.560
Olfactory bulb	4.091 ± 1.411	3.466 ± 1.219

**Table 4:** *Post-hoc* effects of age on [^18^F]FPEB BP in different brain areas (top) and corresponding BP values (below)

Brain area	A1 < A2	A4 < A1	A2 > A3	A3 > A4
	*p*-value	*p*-value	*p*-value	*p*-value
Striatum	3.0 × 10^−5^	2.9 × 10^−17^	1.3 × 10^−14^	1.3 × 10^−5^
Cortex	2.1 × 10^−6^	2.1 × 10^−12^	3.9 × 10^−10^	1.5 × 10^−7^
Hippocampus	2.7 × 10^−7^	4.2 × 10^−17^	6.0 × 10^−12^	8.5 × 10^−11^
Thalamus	1.4 × 10^−4^	5.9 × 10^−12^	5.6 × 10^−13^	7.6 × 10^−3^
Hypothalamus	0.28	2.2 × 10^−11^	1.9 × 10^−5^	1.3 × 10^−3^
Olfactory bulb	2.9 × 10^−8^	1.5 × 10^−2^	1.4 × 10^−5^	9.3 × 10^−5^

	BP_ND_			
	A1	A2	A3	A4
Striatum	5.220 ± 0.997	6.060 ± 0.958	4.380 ± 0.507	3.630 ± 0.552
Cortex	4.870 ± 1.250	6.130 ± 1.280	4.410 ± 0.787	3.210 ± 0.667
Hippocampus	5.620 ± 1.010	6.920 ± 1.190	5.100 ± 0.884	3.660 ± 0.769
Thalamus	3.350 ± 0.488	3.820 ± 0.641	2.860 ± 0.427	2.580 ± 0.373
Hypothalamus	3.100 ± 0.449	3.230 ± 0.501	2.730 ± 0.439	2.410 ± 0.389
Olfactory bulb	3.550 ± 1.440	5.240 ± 1.490	3.940 ± 0.814	2.950 ± 0.728

**Table 5:** *Post-hoc* analysis of interaction effect between age and sex on [^18^F]FPEB BP in different brain areas regardless of disorder status (top) and corresponding BP values at each age (bottom) for male and female groups

Male				
Brain area	A1 < A2	A4 < A1	A2 > A3	A3 > 4
	*p*-value	*p*-value	*p*-value	*p*-value
Striatum	5.5 × 10^−8^	5.5 × 10^−8^	1.3 × 10^−13^	0.012
Hippocampus	1.1 × 10^−8^	5.0 × 10^−6^	6.8 × 10^−10^	7.8 × 10^−5^
Hypothalamus	0.005	2.8 × 10^−4^	1.4 × 10^−5^	0.077

	BP_ND_			
	A1	A2	A3	A4
Striatum	5.136 ± 0.895	6.662 ± 0.733	4.365 ± 0.616	3.798 ± 0.537
Hippocampus	5.333 ± 0.913	7.410 ± 0.948	4.386 ± 0.868	3.426 ± 0.645
Hypothalamus	2.985 ± 0.401	3.423 ± 0.374	5.123 ± 0.993	3.944 ± 0.746

Female				
Brain area	A1 < A2	A4 < A1	A2 > A3	A3 > A4
	*p*-value	*p*-value	*p*-value	*p*-value
Striatum	0.844	4.3 × 10^−12^	7.8 × 10^−4^	8.6 × 10^−5^
Hippocampus	0.169	2.2 × 10^−13^	4.2 × 10^−4^	5.6 × 10^−8^
Hypothalamus	0.177	7.6 × 10^−9^	0.119	0.005

	BP_ND_			
	A1	A2	A3	A4
Striatum	5.295 ± 1.098	5.348 ± 0.661	4.393 ± 0.390	3.464 ± 0.527
Hippocampus	5.877 ± 1.041	6.349 ± 1.232	5.085 ± 0.791	3.387 ± 0.702
Hypothalamus	3.212 ± 0.472	2.992 ± 0.546	2.789 ± 0.308	2.351 ± 0.409

**Table 6:** Age and sex effects on [^18^F]FPEB BP in FMR1-KO mice

	Striatum	Cortex	Hippocampus	Thalamus	Hypothalamus	Olfactory
Sex × Age interaction (*p*-value)	0.006	0.204	0.016	0.438	0.043	0.517

Male				
Brain area	A1 < A2	A4 < A1	A2 > A3	A3 > A4
	*p*-value	*p*-value	*p*-value	*p*-value
Striatum	2.4 × 10^−5^	3.0 × 10^−4^	5.5 × 10^−8^	0.122
Hippocampus	1.5 × 10^−5^	9.8 × 10^−4^	8.9 × 10^−7^	0.018
Hypothalamus	0.013	2.1 × 10^−4^	2.4 × 10^−6^	0.622

	BP_ND_			
	A1	A2	A3	A4
Striatum	5.477 ± 0.971	6.877 ± 0.849	4.543 ± 0.536	3.794 ± 0.609
Hippocampus	5.759 ± 0.875	7.852 ± 0.943	5.720 ± 0.986	4.059 ± 0.860
Hypothalamus	2.908 ± 0.408	3.322 ± 0.355	3.001 ± 0.576	2.575 ± 0.375

Female				
Brain area	A1 < A2	A4 < A1	A2 > A3	A3 > A4
	*p*-value	*p*-value	*p*-value	*p*-value
Striatum	0.560	1.7 × 10^−9^	4.7 × 10^−4^	8.5 × 10^−5^
Hippocampus	0.149	1.7 × 10^−8^	7.7 × 10^−4^	2.2 × 10^−5^
Hypothalamus	0.439	5.9 × 10^−6^	0.129	0.010

	BP_ND_			
	A1	A2	A3	A4
Striatum	5.503 ± 1.361	5.527 ± 0.919	4.619 ± 0.299	3.769 ± 0.393
Hippocampus	6.350 ± 0.904	7.041 ± 1.596	5.721 ± 0.570	3.730 ± 0.589
Hypothalamus	3.256 ± 0.491	2.976 ± 0.754	2.815 ± 0.261	2.524 ± 0.329

## 3 PET results

**Figure 1:**
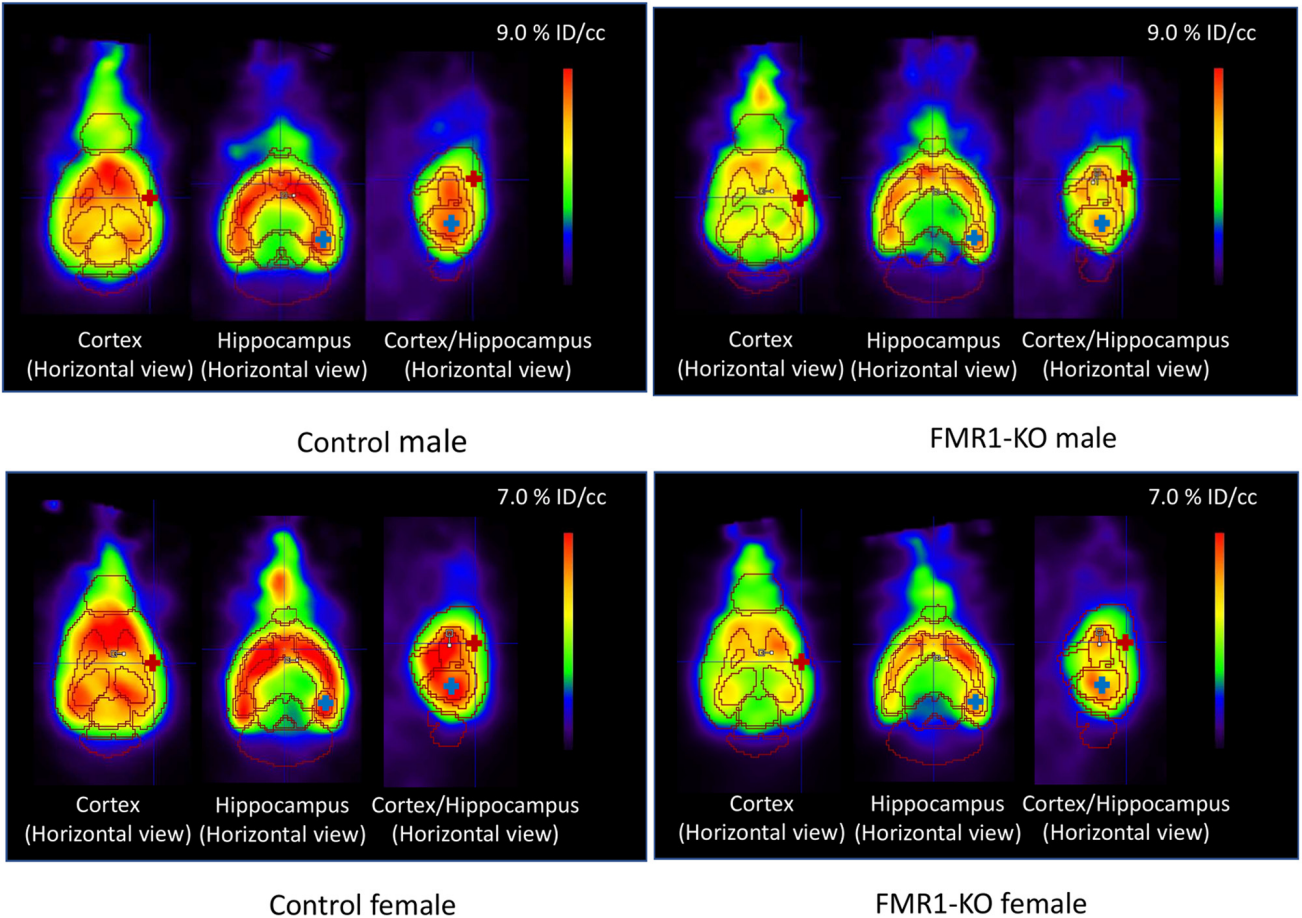
Binding of [^18^F]FPEB to mGluR5 at A2 in male and female mouse from the control and FMR1-KO groups in highlighting significant differences in mGlu5 receptor availability between the groups in key brain areas. [^18^F]FPEB data are averaged in the time window 25–45 min after injection of the radioactivity.

Figure 1 shows the [^18^F]FPEB binding to mGluR5 in one male and one female mouse from each group (FMR1-KO and control); revealing differences in mGluR5 availability between the groups in multiple brain areas known to be impacted in FXS, based on preclinical and clinical studies. We also found changes in [^18^F]FPEB BP related to age and sex, as discussed below. The [^18^F]FPEB uptake in the cerebellum, however, was minimal compared to other brain regions. Importantly, there was no significant difference in cerebellar uptake values between the FMR1-KO and control groups (FMR1-KO: 1.287 ± 0.561% ID/cc and control: 1.345 ± 0.651% ID/cc; *p* = 0.548), further validating the use of this brain area as a reference region. A comparison of [^18^F]FPEB uptake in the cerebellum between male and female mice also revealed no significant difference (male: 1.324 ± 0.614% ID/cc and female: 1.304 ± 0.596% ID/cc; *p* = 0.830).

### 3.1 [^18^F]FPEB BP was significantly lower in FMR1-KO mice than in control mice

**Figure 2:**
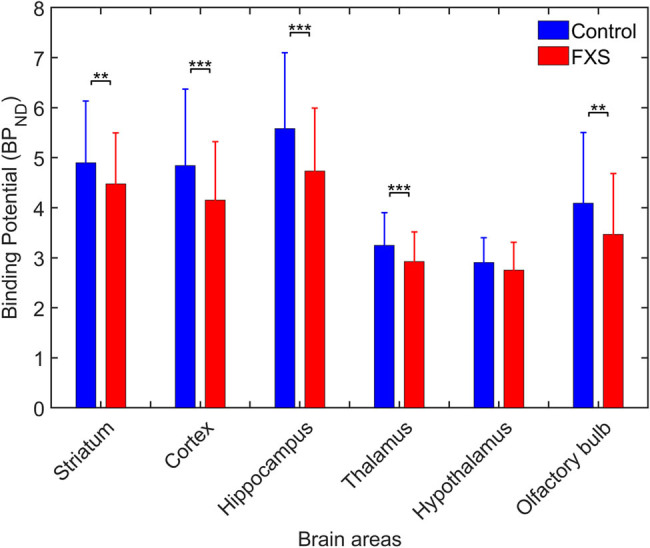
Comparison of [^18^F]FPEB BP_ND_ between FMR1-KO and control mice in different brain areas regardless of age and sex (**p < 0.01; ***p < 0.001) (see Table 3).

Multivariate statistical analysis yielded a main effect for disorder status in all brain areas, *viz*., striatum, cortex, hippocampus, thalamus, and olfactory bulb, except the hypothalamus (Figure 2 and Table 2). BP_ND_ was significantly lower (*p* < 0.008, *corrected*) in FMR1-KO mice than in the control mice in these brain areas, as shown in Table 3. However, there was no significant interaction of disorder status with age or sex in any of the brain areas (Table 2).

### 3.2 [^18^F]FPEB BP declined in later ages in both control and FMR1-KO mice

**Figure 3:**
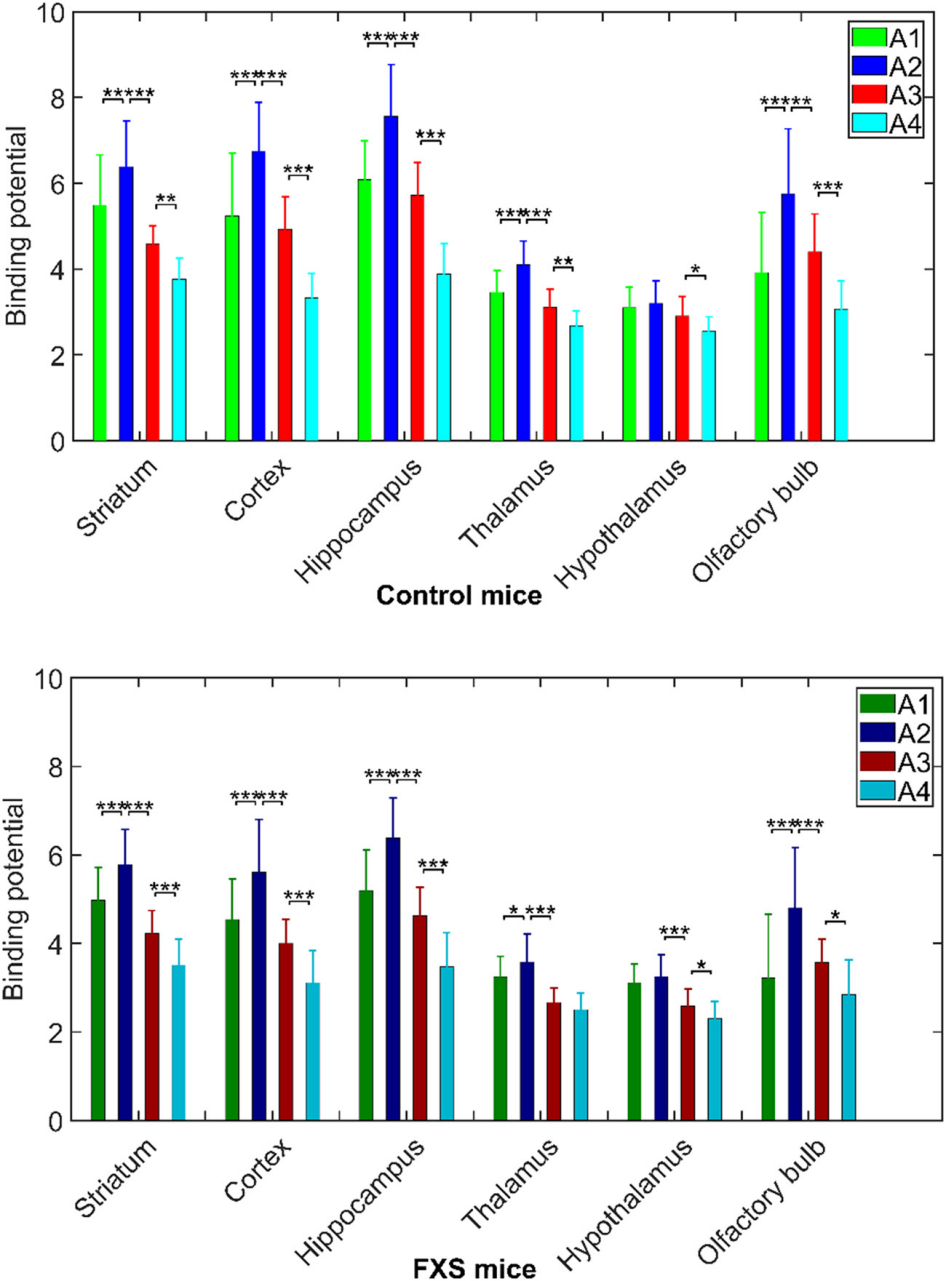
[^18^F]FPEB BP (mean ± SD) in different brain areas as a function of age (A1–A4) in Control group (top) and FMR1-KO group (bottom) (*p < 0.05; **p < 0.01; ***p < 0.001).

We found that age had a significant effect (*p* < 0.008, *corrected*) on [^18^F]FPEB BP in all selected brain areas (Table 2) and the BP_ND_ typically increasing at A2 before declining in later years (Table 4). In both FMR1-KO and control mice (Figure 3), BP changed significantly (*p* < 0.01, *corrected*) with age, increasing from A1 to A2 (except in hypothalamus), and then declined significantly at A3, except in the hypothalamus in control mice, and again at A4, except in the thalamus in the FMR1-KO mice. Whereas there was no age x disorder interaction, age did interact with sex in selected brain regions, as presented below.

### 3.3 [^18^F]FPEB binding to mGluR5 increased from A1 to A2 before falling in selected brain areas, evident in male mice but not in female mice

**Figure 4:**
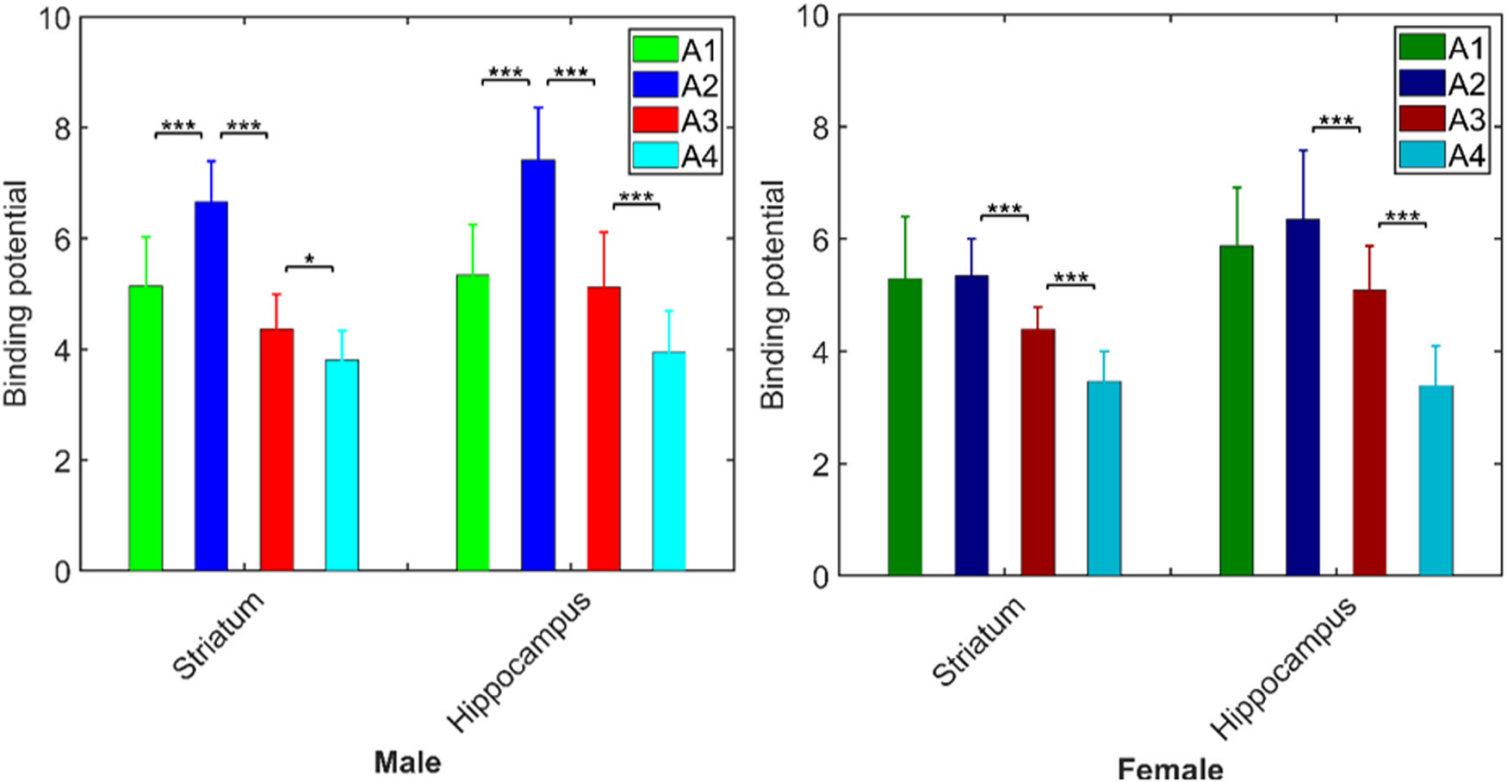
BP as a function of age in male and female mice regardless of genotype (*p < 0.05; ***p < 0.0001).

The linear mixed model analysis of BP revealed a significant interaction between age and sex (*p* < 0.008, *corrected*) in striatum and hippocampus; however, the effect in the hypothalamus did not survive correction for multiple brain region testing (Table 2). The *post-hoc* analysis of interaction in the striatum and hippocampus revealed an increase (*p* < 0.01, *corrected*) in BP from A1 to A2 in male but not in female mice. In contrast, both male and female mouse groups showed a significant decrease in BP in these areas from A2 to A3, and again, from A3 to A4 (though only marginally in the striatum for males; Figure 4 and Table 5 for details).

**Figure 5:**
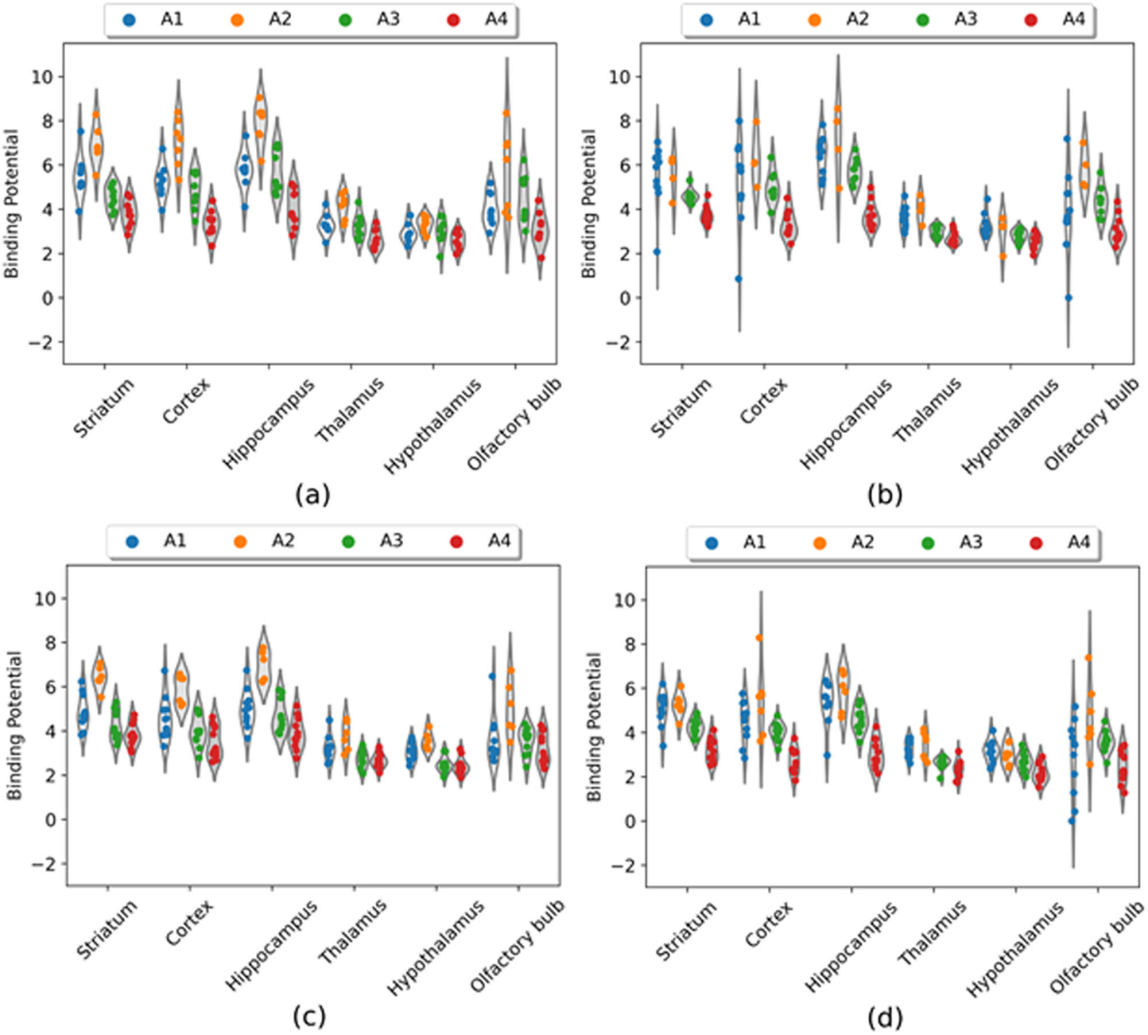
BP in control and FMR1-KO groups as a function of sex and age: (a) control males; (b) control females; (c) FMR1-KO males; and (d) FMR1-KO females.

The exploratory analysis (*p* < 0.05, *uncorrected*) of [^18^F]FPEB BP in the FMR1-KO group alone yielded similar results, *viz*., a significant age by sex interaction in three areas: striatum: *p* = 0.006; hippocampus: *p* = 0.016; and hypothalamus: *p* = 0.043 (Figure 5, Table 6). *Post-hoc* analysis found that FMR1-KO male but not FMR1-KO female mice showed a significant increase in [^18^F]FPEB BP_ND_ from A1 to A2 (Table 6). However, both male and female mice in this group showed a decline in BP_ND_ from A2 to A3 in these areas (except in hypothalamus for females); the significant decline continued at A4 in females (though only marginally in the hypothalamus) but not in males, except marginally in the hippocampus.

## 4 Discussion

Metabotropic glutamate receptors are implicated in the pathogenesis of a variety of neuropsychiatric disorders. Of particular interest in this study were mGlu5 receptors since their exaggerated signaling has been reported to account for the sensory and cognitive impairments that characterize FXS [3,4]. The results of this study confirm the reduced availability of mGlu5 receptors in FXS; using the cerebellum as a reference region, we found significantly lower [^18^F]FPEB BP in the FMR1-KO mice compared to the control mice in key brain areas, consistent with mGluR5 distribution and implicated in the symptomatology of FXS. A reduction in mGluR5 signaling by drugs that block these receptors has reduced in KO mice seizures, auditory hypersensitivity and motor deficits, and improved functional connectivity in sensorimotor networks, though it has been less effective in social and cognitive domains [19]. Other studies have focused on the genetic reduction of mGluR5 as a correcting factor for the behaviors related to fragile X phenotype [3,20]. In general, preclinical studies have confirmed mGluR5 as a valid therapeutic target for the pathophysiology underlying FXS [15,21]. This study of mGluR5, over a one year period, using PET imaging with [^18^F]FPEB in FMR1-KO mice and age- and sex-matched control mice highlights changes in the receptor function with the progression of the disorder which we discuss below.

### 4.1 Reduced [^18^F]FPEB binding to mGluR5 in FMR1-KO mice: a downstream compensatory mechanism?

According to the mGluR theory of FXS, absent or reduced levels of FMRP lead to excessive mGluR5 signaling with potential downstream consequences on receptor availability. In keeping with the mGluR5 theory, we found the reduced [^18^F]FPEB binding in FMR1-KO mice compared to control mice in selected brain areas with known mGluR5 distribution, including striatum, cortex, hippocampus, thalamus, and olfactory bulb. These areas are frequently implicated in the symptoms associated with FXS [22,23,24,25]. However, factors other than decreased mGluR5 availability can contribute to the reduced [^18^F]FPEB binding observed in this study, including increased competition from elevated glutamate levels and changes in blood flow and metabolism. Some studies have found that receptor alignment, positioning, and mobility, critical in receptor signaling and thus its function [26], are altered by fragile-X phenotype progression [27]. The postsynaptic environment of mGlu5 receptors is known to be impacted by the progression of the disorder, affecting receptor signaling and availability [28,29,30,31,32]. Although preliminary, our results are consistent with the mGluR theory of FXS and provide an index of the progression of the disorder with age.

Studies have reported the imbalanced levels of GABA, excitatory and inhibitory neurotransmitters, in subjects with advanced fragile X symptoms [33,34]. Although the level of glutamate was increased in these studies, induced by glutaminase [35], the production of GABA level was found to be insufficient. In fact, the availability of gamma-aminobutyric acid receptor A (GABA_A_) receptors was shown to drop by 17% in the thalamus in subjects with FXS [36]. This reduction is in agreement with the previously reported decreased expression of GABA_A_ receptors in the brain tissue of KO mice [37]). A reduction in the expression of gamma-aminobutyric acid receptor B (GABA_B_) has also been reported in the FMR1-KO mouse [38]. Downregulated inhibitory chemical that modifies GABA effect in the body (GABAergic) signaling induces hyperexcitability both pre- and post-synaptically. The resulting imbalanced levels of glutamate and GABA as well as hyperexcitability lead to neurotoxicity. The induced neurotoxicity and resulting neural cell death can impair the availability of the receptors.

The results of this study are also in keeping with our recent findings in humans, of reduced mGluR5 availability in males with FXS compared to healthy controls [39] as well as those of Brašić and colleagues [40]. In both studies, the availability of the mGlu5 receptors was lower in FXS patients compared to control groups. This significant reduction was found in cingulate cortex, striatum, and thalamus, as well as insula, anterior cingulate, parahippocampal, inferior temporal, and olfactory cortices. Importantly, these areas are associated with behavioral deficits in memory and visuospatial learning, anxiety, and executive functions, characteristic of patients with FXS. However, these results would benefit from examination of competing interpretations toward a robust neurobiological account of the pathophysiology of FXS.

### 4.2 mGlu5 receptor availability in FMR1-KO mouse: effects of age and sex and a potential time window for treatment

PET imaging using [^18^F]FPEB revealed the reduced BP of mGlu5 receptors at older ages in FMR1-KO and control mice. In both groups, BP increased from A1 (1.5–2 month) to A2 (3 month) before it decreased at A3 (5–7 month) and A4 (11–13 month) in all brain areas. However, this finding was modulated by sex in the striatum and hippocampus, which was evident in the KO group when examined separately (Table 6). Specifically, male but not the female mice showed a significant increase in BP at around 2 months of age (A2) in these two regions before BP declined at A3. The decrease in BP at A3 suggests that A2 (3 month) to A3 (5–7 month) may be an optimal time window for intervention particularly for FMR1-KO males who are more severely affected than females with the disorder. That the striatum and hippocampus are involved in motor planning, learning and memory, core areas of deficit in FXS help to strengthen the clinical significance of A2 to A3 as a potential time window for therapeutic intervention. As such, treatment with a drug that inhibits mGluR5 signaling during this critical time window could prevent symptoms from getting worse. The present findings call for longitudinal studies with larger mouse cohorts. Additionally, the female mice in the FMR1-KO group appeared to show an extended window of vulnerability, the BP in this group continuing to fall at A4 (11–13 month), unlike the males for whom BP appeared to level out at A3 (5–7 month). Whereas this finding remains to be verified by future studies, it may suggest a need for additional monitoring of females with FXS especially in their later years. The results of this study also emphasize the importance of early intervention of the disorder [41,42].

These preliminary findings provide potential insights for future studies and tailoring treatments in individuals with FXS. The findings may also inform our understanding of other neurodevelopmental disorders such as autism spectrum disorder, which shares several deficits with FXS in language, learning, and sensorimotor domains [43,44]. The use of PET to examine excitatory and inhibitory neurotransmission profiles of individuals with FXS, with versus without autism, could point to mechanistic differences underpinning these comorbid disorders for more effective treatment targets.

### 4.3 Conclusion and future directions

The use of a longitudinal PET design involving both male and female mice to examine mGluR5 function as it impacts FXS, provides some preliminary but important insights about the disorder. The findings suggest an optimal age, around early adulthood or A2, to target treatment while also highlighting differences between males and females with FXS. Future studies with a larger number of mice comparing the KO mice with their wild-type littermates are essential to interpret the impact of the disorder on both mGlu5 receptors and behavioral performance. Finally, based on the present findings, future studies of humans affected by FXS and FMR1-KO mice may benefit from connectivity analyses that explore subcortical circuitry involving the hippocampus and striatum as potential targets of pharmacological treatment.
